# Efficient out-of-distribution detection via layer-adaptive scoring and early stopping

**DOI:** 10.3389/fdata.2024.1444634

**Published:** 2024-11-20

**Authors:** Haoliang Wang, Chen Zhao, Feng Chen

**Affiliations:** ^1^Department of Computer Science, The University of Texas at Dallas, Richardson, TX, United States; ^2^Department of Computer Science, Baylor University, Waco, TX, United States

**Keywords:** out-of-distribution detection, early stopping, layer-adaptive, deep neural networks, one-class support vector machine

## Abstract

**Introduction:**

Multi-layer aggregation is key to the success of out-of-distribution (OOD) detection in deep neural networks. Moreover, in real-time systems, the efficiency of OOD detection is equally important as its effectiveness.

**Methods:**

We propose a novel early stopping OOD detection framework for deep neural networks. By attaching multiple OOD detectors to the intermediate layers, this framework can detect OODs early to save computational cost. Additionally, through a layer-adaptive scoring function, it can adaptively select the optimal layer for each OOD based on its complexity, thereby improving OOD detection accuracy.

**Results:**

Extensive experiments demonstrate that our proposed framework is robust against OODs of varying complexity. Adopting the early stopping strategy can increase OOD detection efficiency by up to 99.1% while maintaining superior accuracy.

**Discussion:**

OODs of varying complexity are better detected at different layers. Leveraging the intrinsic characteristics of inputs encoded in the intermediate latent space is important for achieving high OOD detection accuracy. Our proposed framework, incorporating early stopping, significantly enhances OOD detection efficiency without compromising accuracy, making it practical for real-time applications.

## 1 Introduction

Deep neural networks (DNNs) have recently shown remarkable performance in classification tasks. However, DNNs are typically trained under the closed-world assumption, which assumes the same data distribution during both training and testing. This assumption poses challenges in real-world applications when encountering data that significantly differs from the training data, known as out-of-distribution (OOD) data. Detecting OOD data is essential because DNNs can produce unreliable or incorrect predictions when faced with such data, especially in safety-critical applications like detecting new object categories in autonomous driving and diagnosing unknown diseases such as COVID-19. Effective OOD detection identifies these data points, enabling the DNN to either reject these inputs or handle them appropriately, thus enhancing the DNN's reliability and robustness.

Many methods have been proposed to detect OOD inputs for DNNs. The majority of these methods detect OOD inputs using predictive uncertainty measures of a softmax classifier, such as entropy (Vyas et al., 2018), epistemic uncertainty (Malinin and Gales, 2018), and others (Hendrycks and Gimpel, 2016; Liang et al., 2017; Zhao and Chen, 2019; Zhao et al., 2021). A more recent work, Deep-MCDD (Lee et al., 2020), estimates a spherical decision boundary for each class based on support vector data description (SVDD). These boundaries enclose the in-distribution (InD) data and distinguish OODs based on their closest class-conditional distribution. Instead of using the last-layer outputs, (Abdelzad et al., 2019) proposed finding the best intermediate layer based on a holdout validation OOD dataset. However, all these methods detect the OOD inputs at the same level of representation (i.e., outputs at a single layer) and fail to account for the different complexity of OOD inputs. Recent studies have shown that multi-layer aggregation is key to the success of OOD detection in DNNs (Ming et al., 2022; Wang et al., 2022; Lambert et al., 2023), indicating that considering a broader spectrum of intermediate layer outputs could lead to more accurate OOD detection than a single-layer solution. This is due to the intrinsic nature of increasingly complex concepts learned at deeper layers in modern DNNs (Zhou et al., 2018). Our empirical study indicates that different OODs are better detected at their appropriate levels of representation (see Section 4.3).

While the effectiveness of OOD detection methods is often the primary focus, in contexts like real-time systems, where resource constraints, scalability, cost-effectiveness, and user experience are crucial, the speed of the OOD detector becomes equally important. With DNNs evolving to process increasingly complex inputs, a practical approach to minimize unnecessary computations involves dynamic depth in inference, which can be facilitated by early stopping strategies. These methods significantly enhance efficiency without compromising, and sometimes even improving, accuracy compared to executing the full network depth (Huang et al., 2017; Kaya et al., 2019; Leroux et al., 2017; Bolukbasi et al., 2017). However, these solutions only focus on InD recognition and cannot be directly applied to OOD detection.

**Figure 1:**
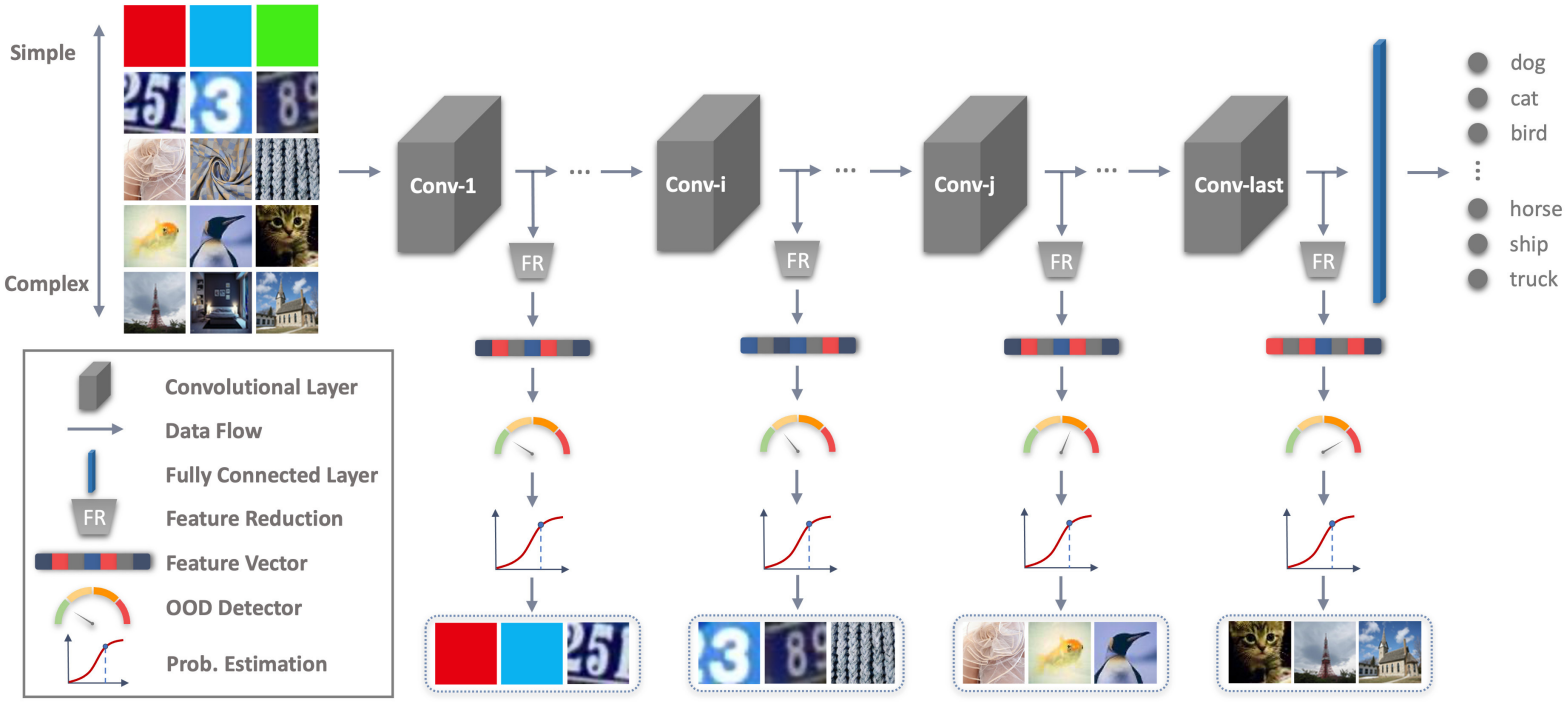
An overview of our proposed Early Stopping OOD Detection (ES-OOD) framework. Input images reproduced with permission from the Tiny ImageNet (https://www.kaggle.com/datasets/akash2sharma/tiny-imagenet), SVHN (https://www.kaggle.com/datasets/stanfordu/street-view-house-numbers), LSUN (https://paperswithcode.com/dataset/lsun) and DTD (https://www.robots.ox.ac.uk/~vgg/data/dtd/) databases.

To address these limitations, we propose the Early Stopping OOD detection (ES-OOD) framework that utilizes all intermediate representations and an early stopping strategy for efficient and effective OOD detection in DNNs (an overview of ES-OOD is shown in Figure 1). Specifically, we train separate One-Class SVM OOD detectors using the outputs of different layers and employ a simple yet effective layer-adaptive scoring function to identify a varying best layer for detecting each potential OOD sample. We enhance the accuracy and robustness of the OOD detectors against unseen OODs by tuning them through self-adaptive data shifting (Wang et al., 2018) and fine-tuning the framework using alternating optimization, which jointly minimizes the DNN classification error and the OOD detectors' training errors. In the test time, our framework adopts “early stopping” to terminate the inference process early when the OOD detectors at intermediate layers provide highly confident OOD predictions. Additionally, ES-OOD uses a voting mechanism to ensure the true positive rate of early-stopped samples. This framework is easy to use and can be applied to any existing DNNs without altering their architectural design.

The main contributions of ES-OOD are as follows:

- We propose a novel Early Stopping OOD detection (ES-OOD) framework that is practical for any off-the-shelf DNNs. Multiple OOD detectors are attached to the intermediate layers of a DNN to fully utilize the intrinsic characteristics of inputs encoded in the intermediate latent space. Through a layer-adaptive scoring function, OODs of varying complexity are detected at their most appropriate layers to achieve higher OOD detection accuracy.
- By integrating an early stopping strategy to detect OODs early and employing a voting mechanism to ensure the true positive rate of the early-stopped samples, we significantly improve efficiency by up to 99.1% while maintaining superior OOD detection efficacy.
- Extensive experiments on three DNNs with varying depth and architectures demonstrate that ES-OOD is robust against OODs of varying complexity and is significantly faster than state-of-the-art baselines while achieving superior OOD detection performance on real-world datasets.

## 2 Related work

### 2.1 Dynamic neural networks with early stopping mechanisms

The concept of early stopping is gaining traction in the field of deep learning. By integrating early exits into DNNs, such systems allow for “simple” samples to be processed at the initial layers, thereby avoiding unnecessary computation (Huang et al., 2017; Kaya et al., 2019). For any given input, an early exit could be triggered by a confidence metric (Leroux et al., 2017) or a learned decision function (Bolukbasi et al., 2017). Traditionally, these methods only focused on improving the performance of DNNs by evaluating InD samples, overlooking OOD samples. *In this paper, we extend the early stopping principle to specifically address the detection of OOD samples, proposing a novel framework designed to efficiently identify “simple” OODs at early layers, thereby conserving computational resources*.

### 2.2 OOD detection for deep neural networks

A main track of recent OOD detection research is to use the final outputs of a DNN to separate OODs from InD samples (Vyas et al., 2018). Hendrycks and Gimpel (2016) proposes a baseline method that detects OODs based on the maximum softmax probabilities of a DNN's final outputs. ODIN Liang et al. (2017) incorporates temperature scaling and input perturbation into the maximum softmax probabilities to enhance the margin between InD and OOD samples. More recently, Lee et al. (2020) extends Deep-SVDD to a multi-class setting and proposes Deep-MCDD, integrating multiple SVDDs into a single deep model, with each SVDD trained to encompass one InD class sample. *However, these methods primarily focus on the high-level features at the final layers of DNNs, potentially neglecting simpler, low-level features at intermediate layers, which might lead to the misclassification of less complex OODs*.

Several studies Abdelzad et al. (2019) and Lee et al. (2018) have explored the use of intermediate outputs for OOD detection. Lee et al. (2018) calculates a confidence score based on a weighted average of the Mahalanobis distance to the nearest class-conditional distribution at each layer, with weights optimized using an additional validation set. Abdelzad et al. (2019) introduces OODL, which identifies an optimal discernment layer based on a holdout OOD dataset. *Despite their innovations, they handle multi-layer aggregation either through a fixed weighting function or by selecting a single “best” layer, limiting their generalizability to unseen OODs*.

Moreover, existing OOD detection techniques typically require the full operation of the neural network to determine if an input is OOD, which is not ideal for real-time applications. *In contrast, our proposed framework, ES-OOD, incorporates an early stopping strategy to significantly reduce computational demands while utilizing outputs from both the final and intermediate layers. This approach enables more precise OOD predictions through layer-adaptive scoring, effectively detecting OOD samples of varying complexity at their most suitable layers*.

## 3 Method

Since OOD samples are rarely available during training, we formulate OOD detection as a one-class classification problem, where OOD detectors determine whether an input is in-distribution or not.

### 3.1 Problem formulation

Consider an input **x** from a set X, with a label *y* from the set Y={1,…,K}, where *K* represents the number of classes. Given a deep neural network M with *L* layers, the network classifies each input into one of *K* classes, denoted as ŷ=M(x)∈Y.

At each layer ℓ, ranging from 1 to *L*, the intermediate output **x**^(ℓ)^ is fed to a layer-specific OOD detector *C*_ℓ_ to compute an OOD score $s^{\left(\ell \right)}=C_{\ell }\left({\text{x}}^{\left(\ell \right)}\right)$. Separate OOD detectors are attached to different layers of M to obtain multiple OOD scores. The final OOD score of **x** can be obtained through a multi-layer aggregation function, such as by taking the average of OOD scores outputted by all OOD detectors:


$$s_{\text{final}}=\frac{1}{L}\overset{L}{\underset{\ell =1}{\sum }}C_{\ell }\left({\text{x}}^{\left(\ell \right)}\right)$$

This final OOD score is then used to determine whether **x** is in-distribution or not based on a predefined threshold δ.

### 3.2 One-class support vector machine

In the domain of one-class classification, numerous techniques can be employed for OOD detection, such as Isolation Forest (iForest; Liu et al., 2008), Gaussian Mixture Models (GMM; Mukhoti et al., 2023), and Autoencoders (Sakurada and Yairi, 2014), among others. In this study, we use the One-Class Support Vector Machine (OCSVM; Schölkopf et al., 2000), a prevalent choice in existing literature due to its effectiveness. The motivation for employing OCSVM stems from its strong theoretical foundation in learning a boundary that encapsulates the majority of the in-distribution data points, making it robust against outliers and suitable for high-dimensional feature spaces. Furthermore, OCSVM is non-parametric, meaning it can model complex distributions without relying on specific assumptions about data structure, which enhances its versatility in detecting diverse OOD samples. It is important to note that our framework design is flexible and allows for the substitution of OCSVM with any other one-class classifiers without altering the underlying architecture.

The OCSVM operates by defining a feature mapping $\Phi :X\subset {\mathbb{R}}^{d}\to F\subset {\mathbb{R}}^{h}$, where *h*>*d*. This mapping transforms input samples ${\left\{{\text{x}}_{i}\right\}}_{i=1}^{n}\in {\mathbb{R}}^{d}$ into a higher dimensional feature space F, aiming to maximize the separation between the input samples and the origin in this space. To achieve this, the OCSVM seeks the optimal hyperplane that maximizes the distance from all input samples to the origin.

The computation typically required for this feature mapping is circumvented using the kernel trick, which computes the inner product in the feature space indirectly. Specifically, we employ the Gaussian Radial Base Function (RBF) kernel for this purpose:


(1) $$\begin{array}{l} k\left({\text{x}}_{i},{\text{x}}_{j}\right)=\exp\left(-\gamma ||{\text{x}}_{i}-{\text{x}}_{j}|{|}^{2}\right), \end{array}$$

Where γ denotes the kernel width. This selection is motivated by the kernel's ability to handle the nonlinear relationships in high-dimensional data effectively.

The optimization of the OCSVM, *C*_ℓ_, at layer ℓ is formulated as a dual Quadratic Programming (QP) problem, which can be solved using Lagrange multipliers. The objective function and constraints are defined as follows:


(2) $$\begin{array}{l} \;\underset{\alpha^{\left(\ell \right)}}{\min}\;\frac{1}{2}\sum_{i,j}\alpha_{i}^{\left(\ell \right)}\alpha_{j}^{\left(\ell \right)}k\left(x_{i}^{\left(\ell \right)},x_{j}^{\left(\ell \right)}\right)\\ \text{s.t. }0\leq \alpha_{i}^{\left(\ell \right)}\leq \frac{1}{\nu n},\text{and }\sum_{i}\alpha_{i}^{\left(\ell \right)}=1 \end{array}$$

Where $\alpha_{i}^{\left(\ell \right)}$ are the Lagrange multipliers and ν∈(0, 1] represents the upper bound of the training error rate.

For an input sample **x** processed at layer ℓ, its OOD score is calculated using the decision function:


(3) $$\begin{array}{l} C_{\ell }\left(\text{x}\right)=-\underset{i}{\sum }\alpha_{i}^{\left(\ell \right)}k\left({\text{x}}_{i}^{\left(\ell \right)},{\text{x}}^{\left(\ell \right)}\right)+\rho^{\left(\ell \right)} \end{array}$$

The offset ρ^(ℓ)^ can be recovered by $\rho^{\left(\ell \right)}=\underset{j}{\sum }\alpha_{j}^{\left(\ell \right)}k\left({\text{x}}_{j}^{\left(\ell \right)},{\text{x}}_{i}^{\left(\ell \right)}\right)$. In this setup, positive OOD scores indicate out-of-distribution samples, whereas negative scores suggest in-distribution samples, assuming a default threshold of zero (δ = 0).

### 3.3 Training procedure

Given a pre-trained DNN model, $M_{\theta }$, parameterized by **θ**, and employing OCSVMs as OOD detectors, we introduce a comprehensive training objective to optimize both the backbone model and the OOD detectors simultaneously. The objective function is defined as follows:


(4) $$\begin{array}{l} \underset{\theta }{\min}\underset{\alpha^{{\left(\ell \right)}_{\ell =1}^{L}}}{\min}\;L(\theta )+\frac{\lambda }{2}\cdot \sum_{\ell =1}^{L}\sum_{i,j}\alpha_{i}^{\left(\ell \right)}\alpha_{j}^{\left(\ell \right)}k\left(x_{i}^{\left(\ell \right)},x_{j}^{\left(\ell \right)}\right)\\ \text{subject to }0\leq \alpha_{i}^{\left(\ell \right)}\leq \frac{1}{\nu n},\text{and }\sum_{i}\alpha_{i}^{\left(\ell \right)}=1 \end{array}$$

This formulation includes two primary components: The first term *L*(***θ***) represents the loss function associated with the DNN, which typically measures the model's accuracy in classifying training data; The second term aggregates the quadratic programming losses from each layer's OCSVM, scaled by a regularization parameter λ>0. Each loss term involves a summation over pairs of data points, weighted by their corresponding Lagrange multipliers $\alpha_{i}^{\left(\ell \right)}$, and computed using a kernel function *k*.

To effectively solve the joint optimization problem defined in Equation 4, we employ an alternating optimization strategy. This technique iteratively updates the model parameters ***θ*** and the dual coefficients ${\left\{\alpha^{\left(\ell \right)}\right\}}_{\ell =1}^{L}$ for the OCSVMs across all layers. The process involves two main steps:

- Step I: Update Model Parameters ***θ***.

In the first step, the dual coefficients ${\left\{\alpha^{\left(\ell \right)}\right\}}_{\ell =1}^{L}$ for each OCSVM are held fixed. The backbone model parameters ***θ*** are then re-estimated by minimizing the following objective:

(5) $$\begin{array}{l} \underset{\theta }{\min}\;L\left(\theta \right)+\frac{\lambda }{2L}\overset{L}{\underset{\ell =1}{\sum }}\underset{i,j}{\sum }\alpha_{i}^{\left(\ell \right)}\alpha_{j}^{\left(\ell \right)}k\left({\text{x}}_{i}^{\left(\ell \right)}\left(\theta \right),{\text{x}}_{j}^{\left(\ell \right)}\left(\theta \right)\right) \end{array}$$

- Step II: Update Dual Coefficients ${\left\{\alpha^{\left(\ell \right)}\right\}}_{\ell =1}^{L}$.

With the updated ***θ*** parameters fixed, the next step is to regenerate the intermediate outputs for the training samples using the current model parameters. These outputs are then used to re-estimate the dual coefficients for each OCSVM by solving the optimization problem outlined in Equation 2.

In training the OCSVMs, two hyperparameters, the Gaussian kernel width γ and the training error upper bound ν, play pivotal roles. γ determines the smoothness of the decision boundary. A smaller γ results in a smoother boundary, which can generalize better but may also underfit by failing to capture finer details in the data distribution. ν on the other hand, sets the maximum allowable training error rate, essentially filtering out noise in the training dataset. It also influences the minimum number of support vectors OCSVM must use, balancing the model's sensitivity to outliers with its ability to detect OOD samples.

Typically, these hyperparameters are optimized using a validation set containing both InD and OOD samples. However, due to the scarcity of OOD training data, we employ self-adaptive data shifting (Wang et al., 2018) for hyperparameter tuning by generating pseudo-OOD samples purely from the available InD data. This allows for a more practical and flexible tuning process, which is particularly beneficial in applications where OOD samples are difficult to collect. Specifically, this approach involves identifying the edge patterns (boundary points) of the target data and shifting them away from the data in the direction of the negative gradient of data density. The magnitude of the shift is chosen such that the pseudo OODs are placed at an optimal distance from the target data, not too far or too close, ensuring proper regulation of the decision boundary without overfitting.

We summarized ES-OOD's training procedure in Algorithm 1.

**Algorithm 1:**
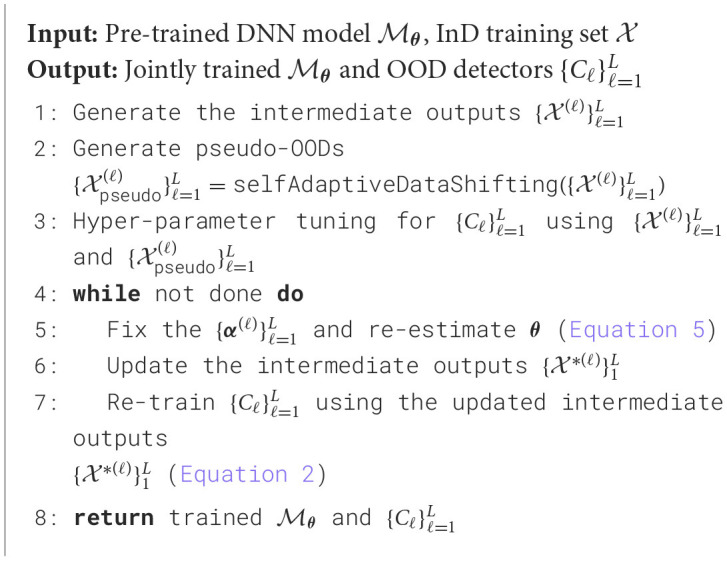
ES-OOD Training procedure.

### 3.4 Layer-adaptive scoring

Given *L* OCSVM OOD detectors ${\left\{C_{\ell }\right\}}_{\ell =1}^{L}$ that each output an OOD score *s*^(ℓ)^ for input **x**, we need to either define a threshold for each OOD detector or design an aggregation function that consolidates all the OOD scores into a final prediction. Empirically, we found that layer-adaptive scoring performs better than a fixed threshold because the predictions of OOD detectors often diverge (see Section **??**). We chose a simple yet effective scoring function that propagates the most confident opinion among all OOD detectors as the final prediction. Specifically, the layer-adaptive scoring is designed as:


(6) $$s_{\text{final}}=\max[{\left\{C_{\ell }(x^{\left(\ell \right)})\right\}}_{\ell =1}^{L}]$$

One challenge with this scoring design is that OCSVMs trained on different features generally produce scores on different scales. This effect can be alleviated by standardizing the training features for each OCSVM: $x^{\prime }=(x-\bar{x})/\sigma$, with $\bar{\text{x}}$ being the sample mean and σ being its standard deviation.

This layer-adaptive scoring is so named because it acts as an equal-weighted (with each layer contributing a weight of 1) maximum OOD score propagation function. It outputs the most confident decision among all the OOD detectors operating at different layers, allowing the model to adaptively choose the most appropriate layer for detecting OOD samples. By applying a threshold to the final OOD scores, OOD samples can be detected at the layer where they receive the highest OOD score. This approach effectively propagates the maximum OOD confidence across layers, ensuring that the most reliable layer is used for detection. As a result, it can utilize the full spectrum of characteristics encoded in different layers to achieve more accurate OOD detection compared to a single-layer solution, underscoring the importance of multi-layer aggregation in the success of OOD detection in DNNs (Ming et al., 2022; Lambert et al., 2023) (see Section 4.6). Meanwhile, unlike other multi-layer aggregation methods (Abdelzad et al., 2019; Lee et al., 2018), it does not require access to any validation data and is robust against unseen OODs (see Section 4.2).

### 3.5 Integrating OOD detection with early stopping

To facilitate early stopping in this OOD detection framework, the most convenient solution is to transform the OOD scores derived from the decision function (Equation 3) into OOD probabilities. By applying a fixed threshold on these probabilities, inputs with high OOD probabilities can be stopped early. Directly applying a fixed threshold on raw OOD scores is infeasible due to scale differences. Alternatively, one can apply layer-specific thresholds on raw OOD scores or on the OOD probabilities to achieve early stopping; however, this solution requires a large validation set and does not generalize well to unseen OODs.

The raw scores from OCSVMs represent distances to the decision boundary, which cannot be directly interpreted as probabilities. To address this, our method involves converting these raw scores into density estimations under the assumption that the raw scores of InD training samples are normally distributed. Specifically, at layer ℓ, we first standardize the raw OOD scores using the mean (μ_ℓ_) and standard deviation (σ_ℓ_) computed from the raw scores of the InD training samples:


(7) $$\begin{array}{l} {\tilde{C}}_{\ell }\left(\text{x}\right)=\frac{C_{\ell }\left(\text{x}\right)-\mu_{\ell }}{\sigma_{\ell }} \end{array}$$

Once standardized, the OOD probability for an input **x** at layer ℓ is estimated using the cumulative distribution function (CDF) of the standard normal distribution:


(8) $$\begin{array}{l} P_{\ell }\left(\text{x}\right)=\int_{-\infty }^{{\tilde{C}}_{\ell }\left(\text{x}\right)}\frac{1}{\sqrt{2\pi }}e^{-\frac{1}{2}t^{2}}dt \end{array}$$

Here, *P*_ℓ_(**x**) represents the probability that input **x** is an OOD at layer ℓ.

By applying a predefined OOD probability threshold 0 <ξ <1 on *P*_ℓ_(**x**), we can implement a simple early stopping strategy during the inference process: If the OOD probability *P*_ℓ_(**x**) exceeds ξ at any layer ℓ, the inference process is terminated, and **x** is identified as OOD. By setting a large threshold ξ, this early stopping strategy can improve OOD inference efficiency by stopping further computation for highly suspected OOD inputs.

Note that instead of assuming a Gaussian distribution for the InD training samples' OOD scores, more sophisticated density estimation methods can be used to achieve better results, such as Kernel Density Estimation (Silverman, 2018), Gaussian Mixture Models (Mukhoti et al., 2023), *k*-Nearest Neighbors (Loftsgaarden and Quesenberry, 1965), or Normalizing Flows (Rezende and Mohamed, 2015). However, fine-tuning the OOD detection accuracy is not the main focus of this work. Our primary focus is the framework design of effective and efficient OOD detection with early stopping. We also demonstrate that even with such a strong assumption, this early stopping strategy can significantly reduce computational overhead while maintaining superior OOD detection performance (see Section 4.2).

### 3.6 Voting mechanism

The early stopping strategy alone is insufficient for effective OOD detection in DNNs due to the accumulation of false positives as the neural network goes deeper. To mitigate this, we integrate a voting mechanism that requires *k*∈ℤ^+^ votes for an input to be identified as OOD and stopped early. Specifically, for an input **x** to be stopped early as OOD, we must have $|{\left\{P_{\ell }\left(\text{x}\right)\right\}}_{1}^{L}>\xi |\geq k$.

This approach significantly lowers the false positive rate, as misclassifying an InD sample as OOD requires multiple detectors at various layers to agree, each surpassing the OOD probability threshold ξ. Although the suitable range of *k* values may vary based on a deep neural network's depth and design, it can be easily determined by a small validation set. Our findings suggest that under appropriate settings, ES-OOD is robust to variations in *k* (see Section 4.4).

The complete inference procedure is summarized in Algorithm 2.

**Algorithm 2:**
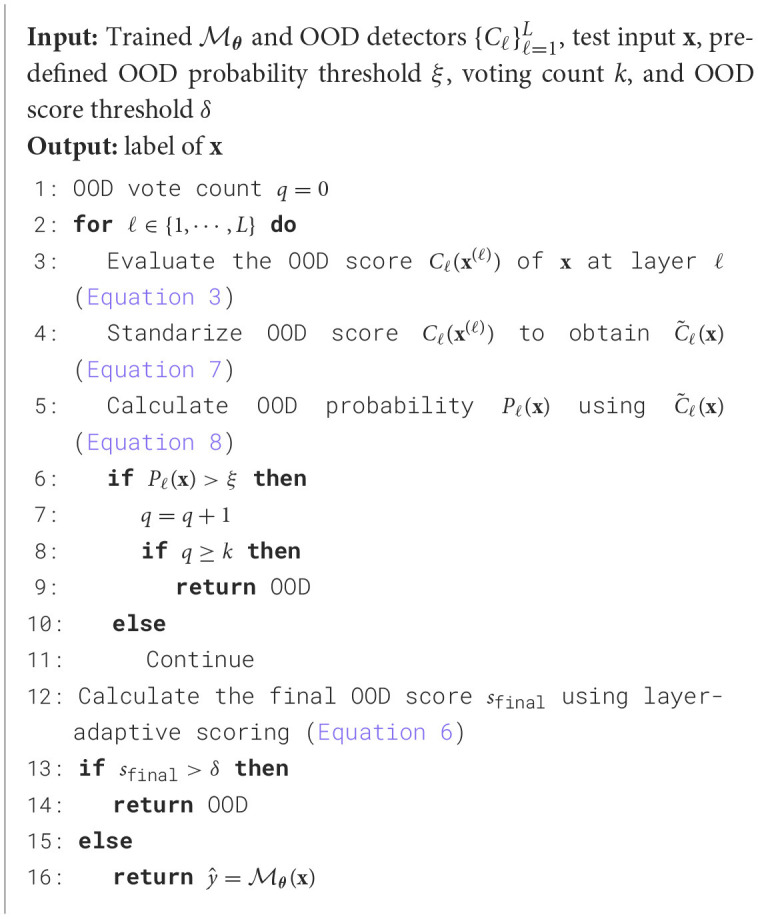
ES-OOD Inference procedure.

## 4 Experiments

### 4.1 Experimental settings^1^

We evaluated ES-OOD on its OOD detection accuracy and efficiency by utilizing two common InD datasets, five OOD datasets of varying complexity, and three widely used DNN models. This comprehensive testing setup was compared against four SOTA baselines to demonstrate the effectiveness and efficiency of our approach.

#### 4.1.1 Datasets

We utilized two InD datasets (CIFAR10 and CIFAR100) and five OOD datasets (LSUN, Tiny ImageNet, SVHN, DTD; Cimpoi et al., 2014, and Pure Color) in our experiments. The “Pure Color” dataset is a synthetic dataset that contains 10,000 randomly generated pure-color images. For each InD-OOD combination, we construct a training set using all the training images in the InD dataset and form a balanced test set using all the test images in both InD and OOD datasets. If the test set is unequal in size, we randomly select images from the larger set to match the size of the smaller one. All images were downsampled to a resolution of 32 × 32 using Lanczos interpolation.

It is important to note that while prior studies often used linear interpolation for downsampling (Abdelzad et al., 2019; Lee et al., 2020, 2018; Liang et al., 2017), *we found that using linear interpolation will introduce severe aliasing artifacts, making such OOD samples easily detectable*. To address this, we employed Lanczos interpolation for downsampling OOD images, a more sophisticated technique that better preserves the original image quality and ensures more authentic OOD samples.

#### 4.1.2 Backbone models

Our evaluation employed three widely recognized DNNs in computer vision and machine learning: VGG-16, ResNet-34, and DenseNet-100. These models, which vary in depth and architecture, demonstrate the adaptability of our framework. All models were trained in traditional image classification settings using stochastic gradient descent (SGD) with a momentum of 0.9. Specifically, VGG-16 was trained for 300 epochs with a batch size of 128, using weight decay regularization of 5 × 10^−4^ and an initial learning rate of 0.05, which was halved every 30 epochs. ResNet and DenseNet followed the training settings detailed in Lee et al. (2018, 2020). The image classification accuracies of these models are presented in Table 1.

**Table 1:** Image classification accuracy of the trained backbone models.

**Model**	**CIFAR10**	**CIFAR100**
VGG-16	93.94%	74.13%
ResNet-34	94.67%	75.02%
DenseNet-100	95.06%	77.18%

#### 4.1.3 Feature reduction

We implemented a feature reduction step on the intermediate outputs to ensure scalability (Abdelzad et al., 2019). Among the various pooling methods tested (including max/average pooling of different sizes and global max/average pooling), global average pooling was found to be the most effective. The resulting features were then standardized using the mean and standard deviation of the training set, as detailed in Section 3.4.

#### 4.1.4 Hyperparameters tuning

For OCSVM, we set the parameter ν at 0.001 to minimize the number of InD samples allowed to be noise. The parameter γ is optimized using pseudo-OODs, created by self-adaptive data shifting of InD training samples only (Wang et al., 2018). The search range for γ is [0.001, 0.0025, 0.005, 0.01, 0.025, 0.05, 0.1, 0.25, 0.5, 1.0], which can be further narrowed based on the specific InD-backbone combination to suit feature complexity and reduce training time. For the early stopping strategy, following (Lee et al., 2018), a validation set of 500 randomly selected OOD samples was used to determine the voting count *k*. We set the OOD probability threshold ξ as 0.99 to ensure a low false positive rate and search for *k* within 20–70% of a DNN's total length, aligning it with specific architectural design.

#### 4.1.5 Baseline methods and evaluation metrics

We benchmark our method against four SOTA OOD detection techniques: MSP (Hendrycks and Gimpel, 2016), ODIN (Liang et al., 2017) (employing both temperature scaling and input preprocessing for optimal performance), OODL (Abdelzad et al., 2019) (with iSUN (Xu et al., 2015) as an additional OOD dataset to determine the optimal discernment layer), and Deep-MCDD (Lee et al., 2020). For evaluating the OOD detection effectiveness, we utilize three common metrics: AUROC, AUPR, and FPR at 95% TPR. To evaluate efficiency, we calculate the percentage of total FLOPS (floating point operations executed per second) saved by the early stopping strategy.

### 4.2 Performance evaluation

Without considering the early stopping, a performance comparison is reported in Table 2. The mean values of each evaluation metric are also included to demonstrate the overall performance on OOD datasets with varying complexity.

**Table 2:** Performance evaluation without early stopping.

**InD/model**	**OOD**	**AUROC ↑**	**AUPR ↑**	**FPR at 95% TPR ↓**
**MSP/ODIN/Deep-MCDD/OODL/ES-OOD(ours)**				
Cifar10 VGG-16	LSUN	86.25 / 86.75 / 85.19 / **88.03** / 87.98	85.26 / 87.06 / 84.76 / **88.01** / 85.31	69.27 / 67.72 / 59.09 / 62.38 / **54.75**
		Tiny	85.66 / 86.35 / 83.95 / 87.10 / **88.64**	84.23 / 86.22 / 83.49 / 86.98 / **87.21**	67.36 / 64.30 / 61.56 / 64.08 / **45.99**
		SVHN	91.12 / 91.47 / 89.81 / 91.68 / **95.17**	87.06 / 89.29 / 93.99 / 88.46 / **94.68**	21.78 / 25.45 / 64.02 / 23.52 / **17.37**
		DTD	87.73 / 90.26 / 88.33 / 92.16 / **97.29**	87.05 / 89.58 / 80.60 / 90.82 / **97.49**	66.24 / 46.33 / 53.56 / 25.04 / **14.06**
		Pure color	98.57 / 99.77 / 98.42 / 99.41 / **99.96**	98.18 / 99.75 / 98.30 / 98.94 / **99.87**	04.66 / 01.24 / 05.68 / 02.08 / **00.13**
		**Mean**	89.87 / 90.92 / 89.14 / 91.68 / **93.81**	88.36 / 90.38 / 88.23 / 90.64 / **92.91**	45.86 / 41.01 / 48.78 / 35.42 / **26.46**
Cifar100 VGG-16	LSUN	73.00 / 73.58 / 72.83 / **75.10** / 72.48	68.49 / 69.78 / **69.92** / 69.68 / 65.28	75.43 / **74.92** / 85.12 / 74.99 / 80.24
		Tiny	77.10 / 77.83 / 76.37 / 79.84 / **80.57**	72.64 / 74.82 / 73.27 / **75.20** / 75.19	63.53 / 68.89 / 80.50 / 60.68 / **56.22**
		SVHN	75.43 / 78.18 / 74.98 / 78.43 / **87.07**	71.53 / 76.20 / **86.52** / 72.63 / 85.82	66.26 / 70.29 / 82.31 / 62.78 / **48.94**
		DTD	75.75 / 76.81 / 73.80 / 77.76 / **93.28**	70.20 / 72.94 / 58.84 / 70.63 / **93.33**	62.13 / 64.66 / 82.20 / 57.82 / **33.20**
		Pure color	62.66 / 51.22 / 78.28 / 58.10 / **96.71**	54.24 / 49.93 / 73.44 / 49.13 / **95.24**	72.32 / 95.31 / 81.83 / 64.85 / **30.08**
		**Mean**	72.79 / 71.52 / 75.25 / 73.85 / **86.02**	67.42 / 68.73 / 72.40 / 67.45 / **82.97**	67.93 / 74.81 / 82.39 / 64.22 / **49.74**
Cifar10 ResNet-34	LSUN	90.16 / 90.26 / 88.02 / **91.97** / 89.06	87.62 / 90.19 / 86.74 / **90.56** / 84.48	33.24 / 50.28 / 55.75 / **31.19** / 37.35
		Tiny	86.53 / 85.46 / 83.34 / 88.81 / **89.29**	84.79 / 86.46 / 83.25 / **87.66** / 86.47	58.26 / 74.41 / 61.28 / 46.15 / **36.90**
		SVHN	84.33 / 81.22 / 88.08 / 87.74 / **97.77**	81.88 / 81.89 / 93.97 / 85.13 / **97.67**	66.58 / 81.16 / 57.06 / 42.84 / **12.17**
		DTD	87.64 / 83.96 / 84.56 / 92.10 / **97.91**	85.24 / 84.39 / 75.07 / 91.10 / **98.06**	51.61 / 78.01 / 62.13 / 30.82 / **11.84**
		Pure color	94.59 / 96.84 / 96.11 / 95.52 / **99.99**	93.48 / 96.93 / 93.81 / 94.35 / **99.99**	17.84 / 15.54 / 36.80 / 19.50 / **00.04**
		**Mean**	88.65 / 87.55 / 88.02 / 91.23 / **94.80**	86.60 / 87.97 / 86.57 / 89.76 / **93.33**	45.51 / 59.88 / 54.60 / 34.10 / **19.66**
Cifar100 ResNet-34	LSUN	75.63 / **77.52** / 74.65 / 51.91 / 65.25	70.76 / **72.81** / 70.14 / 51.92 / 59.65	**62.63** / 63.51 / 84.34 / 94.84 / 78.61
		Tiny	78.70 / **81.28** / 78.29 / 67.05 / 75.82	74.47 / 77.39 / **78.26** / 66.91 / 73.74	57.97 / **57.47** / 78.84 / 90.27 / 68.91
		SVHN	78.76 / 84.16 / 78.62 / 79.00 / **84.61**	73.71 / 78.74 / **88.50** / 69.18 / 76.09	55.29 / 46.58 / 77.50 / 45.81 / **36.85**
		DTD	75.32 / 78.94 / 77.11 / 86.25 / **91.39**	70.07 / 74.52 / 84.85 / 83.45 / **91.97**	62.59 / 60.60 / 81.49 / **40.94** / 41.19
		Pure color	55.23 / 62.25 / 63.47 / 96.46 / **99.80**	48.09 / 52.11 / 53.16 / 91.14 / **99.78**	67.52 / 59.04 / 99.32 / 04.98 / **01.04**
		**Mean**	72.73 / 76.83 / 74.43 / 76.13 / **83.37**	67.42 / 71.11 / 74.98 / 72.52 / **80.25**	61.20 / 57.44 / 84.30 / 55.37 / **45.32**
Cifar10 DenseNet-100	LSUN	92.07 / **94.01** / 87.19 / 88.47 / 84.38	89.47 / **93.12** / 86.23 / 84.87 / 80.95	26.40 / **23.71** / 55.00 / 40.69 / 51.55
		Tiny	89.96 / **91.95** / 85.22 / 84.62 / 88.75	87.69 / **91.32** / 84.44 / 80.90 / 87.80	35.09 / **34.04** / 58.14 / 57.25 / 43.73
		SVHN	89.00 / 89.54 / 89.48 / 97.19 / **97.79**	85.73 / 88.11 / 94.46 / **97.54** / 97.51	36.33 / 43.54 / 51.29 / 16.07 / **09.41**
		DTD	88.65 / 85.42 / 86.93 / 95.10 / **97.61**	86.06 / 84.75 / 77.33 / 96.14 / **97.58**	39.61 / 60.98 / 59.57 / 33.07 / **12.00**
		Pure color	91.83 / 96.78 / 96.21 / 79.15 / **99.97**	87.80 / 95.01 / 95.08 / 69.92 / **99.97**	16.06 / 09.31 / 23.84 / 40.08 / **00.17**
		**Mean**	90.30 / 91.54 / 89.01 / 88.91 / **93.70**	87.35 / 90.46 / 87.51 / 85.87 / **92.76**	30.70 / 34.32 / 49.57 / 37.43 / **23.37**
Cifar100 DenseNet-100	LSUN	76.38 / **77.41** / 75.17 / 59.11 / 69.69	72.14 / **73.19** / 71.18 / 57.10 / 64.28	**62.62** / 65.02 / 82.93 / 91.64 / 72.59
		Tiny	79.73 / **84.27** / 78.25 / 61.84 / 81.29	76.10 / **81.66** / 75.11 / 59.22 / 78.81	55.24 / **50.97** / 77.48 / 81.85 / 62.76
		SVHN	80.08 / 81.30 / 74.99 / 71.73 / **86.99**	75.29 / 74.89 / **86.25** / 65.36 / 78.23	51.73 / 49.32 / 82.48 / 66.07 / **32.89**
		DTD	73.18 / 70.29 / 79.34 / 84.69 / **93.79**	69.03 / 67.93 / 66.09 / 84.72 / **93.95**	73.09 / 91.60 / 75.11 / 56.15 / **30.67**
		Pure color	79.60 / 80.86 / 91.14 / 85.39 / **99.47**	73.54 / 77.68 / 89.64 / 79.53 / **99.41**	44.87 / 61.26 / 49.77 / 34.72 / **02.84**
		**Mean**	77.79 / 78.83 / 79.78 / 72.55 / **86.25**	73.22 / 75.07 / 77.65 / 69.19 / **82.94**	57.51 / 63.63 / 73.55 / 66.09 / **40.35**

Evaluation metrics with “↑” indicate higher values are better, while those with “↓” indicate lower values are better. The best values are highlighted in **bold**.

It can be observed that OODs of higher complexity are harder to detect, such as LSUN and Tiny ImageNet images, which may contain complex backgrounds or multiple objects. In contrast, OODs of lower complexity are easier to detect, such as SVHN, which contains cropped street view house numbers, or DTD, which contains images of different textures. The synthetic Pure Color dataset is of the lowest complexity, as it contains limited information.

Furthermore, single-layer solutions are not robust against all OODs of different complexity. OOD detection methods that rely on features from the final layers (MSP, ODIN, and Deep-MCDD) tend to perform well with complex OODs like LSUN and Tiny ImageNet. However, these methods struggle with simpler OODs such as SVHN, DTD, and Pure Color. The OODL baseline, which leverages a fixed best layer, shows a similar performance pattern to MSP, ODIN, and Deep-MCDD, primarily because LSUN and Tiny ImageNet are of similar complexity as the iSUN dataset, which was used for calibrating OODL's best layers. When the complexity of test OODs differs significantly from iSUN, OODL's performance deteriorates significantly.

Through multiple intermediate OOD detectors and the layer-adaptive scoring, ES-OOD exploits the full-spectrum characteristics encoded in different latent spaces. By considering the early layers' outputs, ES-OOD significantly outperforms the four baseline methods on OOD datasets of lower complexity (SVHN, DTD, and Pure Color). More importantly, ES-OOD achieves the best average AUROC, AUPR, and FPR at 95% TPR for all InD-Backbone settings, demonstrating its robustness against OODs of varying complexity. Overall, ES-OOD shows an 8.21% improvement in AUROC, a 7.8% improvement in AUPR, and a 29.98% improvement in FPR at 95% TPR compared to the second-best baseline method.

If taking the early stopping into consideration, we compared the OOD detection performance and efficiency gain of ES-OOD with or without the Early Stopping (ES) in Table 3. Efficiency gain refers to the computational savings in terms of FLOPs (Floating Point Operations), which are reduced by halting the OOD inference process early when a confident decision is made. ES-OOD with early stopping (w/ ES) significantly reduces OOD inference time while maintaining comparable or even better OOD detection performance. Overall, integrating early stopping improves general inference time by 18.9–65.1%, and by up to 99.1% for simpler OODs like DTD and Pure Color, demonstrating substantial efficiency improvement while preserving high OOD detection accuracy.

**Table 3:** Performance comparison of ES-OOD with (w/) or without (w/o) Early Stopping (ES).

**InD/model**	**OOD**	**AUROC** ↑			**AUPR** ↑			**FPR at 95% TPR** ↓			**Efficiency gain (%) ↑**
		**w/o ES**	**w/ ES**	**Diff**	**w/o ES**	**w/ ES**	**Diff**	**w/o ES**	**w/ ES**	**Diff**	
Cifar10 VGG-16	LSUN	**87.98**	87.61	-0.37	**85.31**	84.79	-0.52	**54.75**	54.75	0.00	3.83
		Tiny	88.64	**88.76**	0.12	87.21	**87.85**	0.64	45.99	**45.99**	-0.00	12.32
		SVHN	95.17	**96.54**	1.37	94.68	**96.77**	2.09	17.37	**16.95**	-0.42	62.78
		DTD	**97.29**	97.22	-0.07	**97.49**	97.39	-0.10	14.06	**13.30**	-0.76	72.85
		Pure color	**99.96**	99.26	-0.70	**99.87**	99.28	-0.59	**00.13**	01.40	1.27	97.85
		**Mean**	93.81	**93.88**	0.07	92.91	**93.22**	0.31	**26.46**	26.48	0.02	49.93
Cifar100 VGG-16	LSUN	**72.48**	72.01	-0.47	65.28	**65.38**	0.10	80.24	**80.00**	-0.24	2.76
		Tiny	**80.57**	80.47	-0.10	75.19	**77.04**	1.85	56.22	**56.16**	-0.06	13.34
		SVHN	87.07	**87.21**	0.14	85.82	**87.02**	1.20	48.94	**48.89**	-0.05	33.80
		DTD	93.28	**93.36**	0.08	93.33	**93.78**	0.45	33.20	**32.22**	-0.98	68.85
		Pure color	96.71	**98.44**	1.73	95.24	**98.49**	3.25	30.08	**02.95**	-27.13	98.56
		**Mean**	86.02	**86.30**	0.28	82.97	**84.34**	1.37	49.74	**44.04**	-5.70	43.46
Cifar10 ResNet-34	LSUN	**89.06**	85.63	-3.43	**84.48**	81.20	-3.28	**37.35**	37.69	0.34	16.38
		Tiny	**89.29**	88.42	-0.87	86.47	**87.23**	0.76	**36.90**	37.03	0.13	36.04
		SVHN	**97.77**	96.96	-0.81	**97.67**	97.04	-0.63	12.17	**11.87**	-0.30	86.61
		DTD	**97.91**	96.63	-1.28	**98.06**	96.78	-1.28	**11.84**	13.35	1.51	87.11
		Pure color	**99.99**	98.23	-1.76	**99.99**	98.29	-1.70	**00.04**	03.36	3.32	99.09
		**Mean**	**94.80**	93.17	-1.63	**93.33**	92.11	-1.22	**19.66**	20.66	1.00	65.05
Cifar100 ResNet-34	LSUN	65.25	**65.47**	0.22	59.65	**65.27**	5.62	78.61	**78.56**	-0.05	4.76
		Tiny	**75.82**	74.81	-1.01	73.74	**76.00**	2.26	68.91	**68.91**	-0.00	12.18
		SVHN	84.61	**85.00**	0.39	76.09	**85.44**	9.35	**36.85**	37.65	0.80	13.37
		DTD	**91.39**	87.10	-4.29	**91.97**	88.73	-3.24	**41.19**	41.24	0.05	36.83
		Pure color	**99.80**	91.30	-8.50	**99.78**	92.59	-7.19	**01.04**	16.53	15.49	64.68
		**Mean**	**83.37**	80.74	-2.63	80.25	**81.61**	1.36	**45.32**	48.58	3.26	26.36
Cifar10 DenseNet-100	LSUN	**84.38**	84.34	-0.04	80.95	**82.43**	1.48	51.55	**51.51**	-0.04	17.29
		Tiny	**88.75**	88.65	-0.10	87.80	**88.56**	0.76	**43.73**	43.78	0.05	35.26
		SVHN	**97.79**	96.97	-0.82	**97.51**	97.10	-0.41	09.41	**04.93**	-4.48	71.86
		DTD	**97.61**	96.38	-1.23	**97.58**	96.57	-1.01	12.00	**08.63**	-3.37	74.22
		Pure color	**99.97**	97.50	-2.47	**99.97**	97.62	-2.35	**00.17**	04.74	4.57	88.26
		**Mean**	**93.70**	92.77	-0.93	**92.76**	92.46	-0.30	23.37	**22.72**	-0.65	57.38
Cifar100 DenseNet-100	LSUN	69.69	**69.92**	0.23	64.28	**69.55**	5.27	**72.59**	72.64	0.05	5.64
		Tiny	**81.29**	79.85	-1.44	78.81	**80.43**	1.62	**62.76**	63.12	0.36	11.21
		SVHN	86.99	**89.85**	2.86	78.23	**90.86**	12.63	**32.89**	33.29	0.40	18.10
		DTD	**93.79**	90.27	-3.52	**93.95**	91.40	-2.55	30.67	**30.44**	-0.23	25.32
		Pure color	**99.47**	93.14	-6.33	**99.41**	93.97	-5.44	**02.84**	13.03	10.19	34.08
		**Mean**	**86.25**	84.61	-1.64	82.94	**85.24**	2.30	**40.35**	42.50	2.15	18.87

The “Diff” columns are calculated by “w/ ES” minus “w/o ES.” The green shading indicates that “w/ ES” achieved better results, while red shading indicates the opposite. Evaluation metrics with “↑” indicate higher values are better, while those with “↓” indicate lower values are better. The best values are highlighted in bold.

### 4.3 Layer-specific OOD detection dynamics

**Figure 2:**
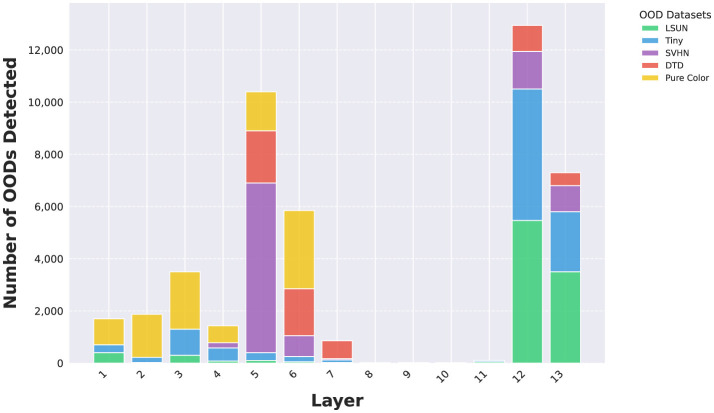
Number of OODs detected by OOD detectors at different layers using VGG-16 and CIFAR10 InD.

As DNN layers deepen, they learn increasingly complex features (Zhou et al., 2018). By equipping intermediate layers with OOD detectors, we can identify OODs based on varying feature complexity. Using the VGG model and CIFAR10 InD as an example, Figure 2 illustrates the number of OODs detected by each OOD detector. For more complex OODs like LSUN and Tiny ImageNet, the majority are identified by the last two OOD detectors. Conversely, simpler OODs are primarily detected by the first seven OOD detectors.

**Figure 3:**
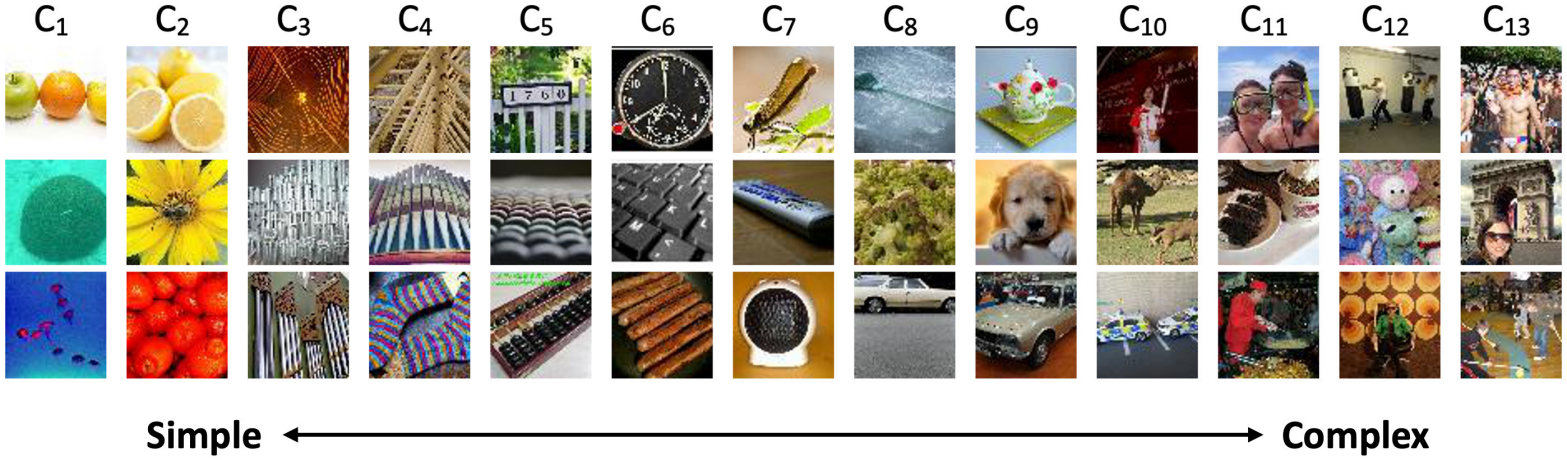
Correctly identified Tiny ImageNet OODs by OOD detectors at different layers, using VGG backbone and CIFAR10 as InD dataset. Input images reproduced with permission from the Tiny ImageNet (https://www.kaggle.com/datasets/akash2sharma/tiny-imagenet) database.

**Figure 4:**
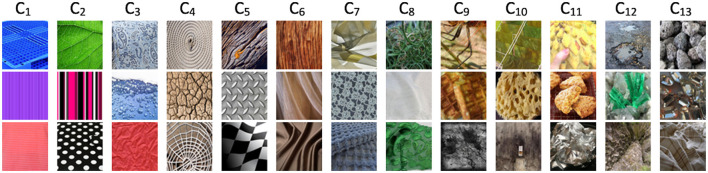
Correctly identified DTD OODs by OOD detectors at different layers, using VGG backbone and CIFAR10 as InD dataset. Input images reproduced with permission from the DTD (https://www.robots.ox.ac.uk/~vgg/data/dtd/) database.

Figure 3 illustrates the Tiny ImageNet OODs detected by OOD detectors at various layers using the VGG backbone and CIFAR10 as the InD dataset. Detectors in the early layers are more sensitive to image colors and textures, capturing fine-scale details. In contrast, detectors in the final layers tend to identify OODs based on objects or scenes, demonstrating their ability to recognize more complex OODs. A similar pattern is observed for the DTD dataset, as shown in Figure 4.

### 4.4 Advantages of using intermediate OOD detectors

**Figure 5:**
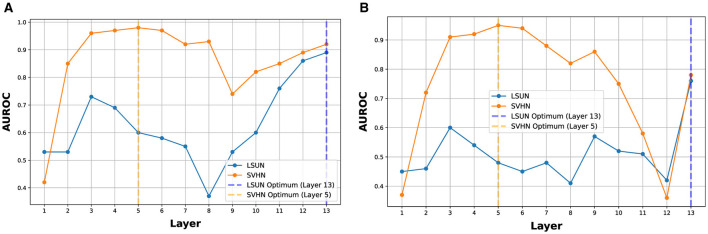
The optimal discernment layers of SVHN and LSUN on VGG-16. **(A)** Optimal discernment layer when CIFAR10 as InD. **(B)** Optimal discernment layer when CIFAR100 as InD.

An optimal discernment layer (or best layer) can be found for a particular OOD dataset, but it may not be the optimal choice for OOD datasets of different complexity (Abdelzad et al., 2019). In Figure 5, we show the AUROC of SVHN and LSUN at each layer of VGG-16 (using CIFAR10 and CIFAR100 as InD, respectively). The best layer for SVHN is layer 5, while the best layer for LSUN is the last layer. Such a best layer could be estimated using a separate OOD dataset; however, as observed in Table 2, OODL that estimates the best layer using the iSUN dataset can have its performance degrade significantly when encountering OODs of different complexity. Therefore, instead of choosing the best layers for different OODs, ES-OOD propagates the most confident OOD prediction across all layers and can effectively construct a good OOD confidence measurement for unseen OODs. For all five OOD datasets considered in this paper, ES-OOD can achieve competitive or even better accuracy compared to their corresponding best layers (see Section 4.6).

### 4.5 Performance-cost trade-off of using early stopping: a sensitivity snalysis

Two critical hyperparameters for early stopping are the OOD probability threshold ξ, which determines when an input is flagged as OOD at each layer, and the voting count *k*, which sets the minimum number of votes an input must receive across layers to determine its final identity (OOD or not). Both hyperparameters control the performance-cost trade-off in different ways.

To analyze the effect of the OOD probability threshold ξ, let's assume all the OOD detectors at different layers are well-trained and can produce reasonably accurate OOD probability estimations for the inputs. In this case, OODs will generally have higher OOD probabilities than InDs, hence, a higher OOD probability threshold ξ can potentially reduce the false positive rate as fewer InDs will be misclassified as OOD. However, a higher OOD probability threshold ξ will also lead to fewer inputs being identified early as OODs, requiring more computational resources.

**Figure 6:**
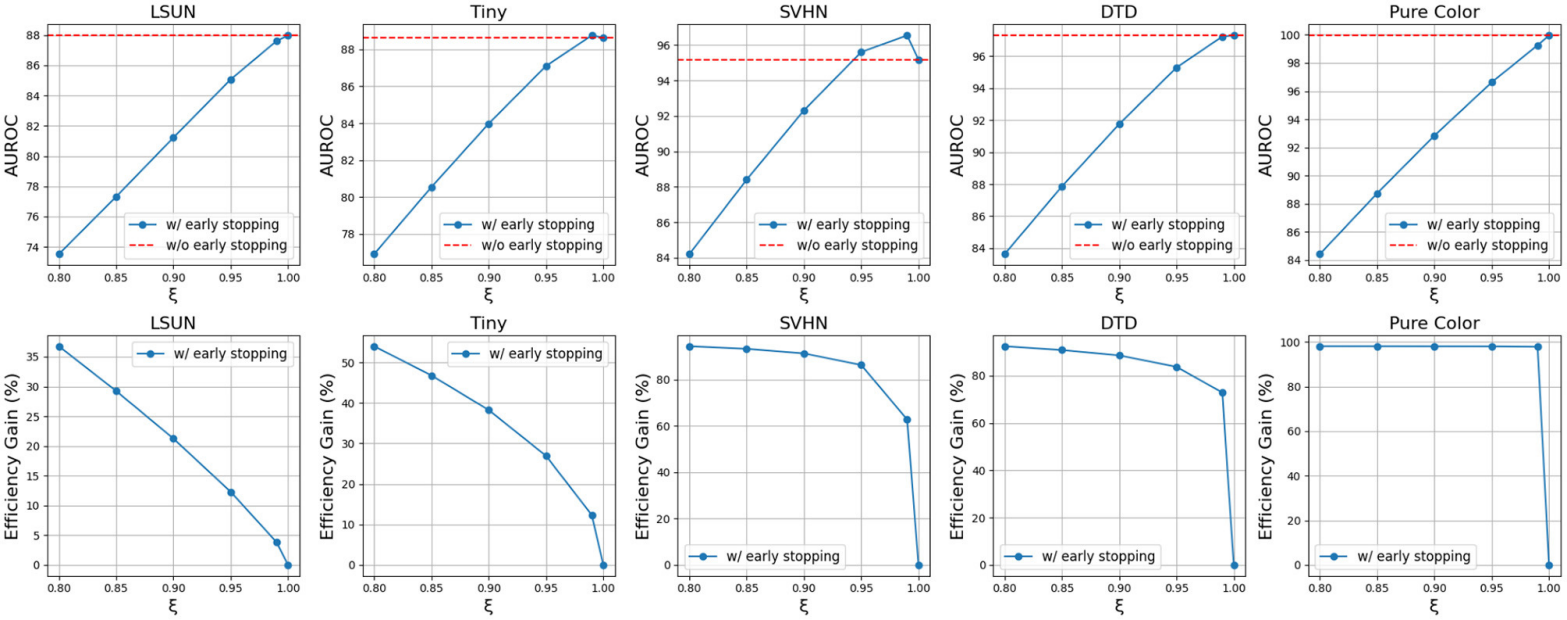
Trends of AUROC and efficiency gain as OOD probability threshold ξ increase using VGG-16 backbone and CIFAR10 InD.

Using the CIFAR10-VGG16 setting as an example, as shown in Figure 6, as ξ increases from 0.80 to 0.99, OOD detection accuracy increases while efficiency gain reduces for all OODs. However, as ξ increases from 0.99 to 1.0, the OOD detection accuracy either drops or increases slightly, while the efficiency gain reduces to zero as no OOD will stop early. This means early stopping can sometimes help increase OOD detection performance while reducing the inference time. Depending on the application, the suitable ξ range may vary according to its priority in performance and cost, but for general applications, it is reasonable to set 0.95 ≤ ξ <1.0 to maintain high OOD detection accuracy while being more efficient. From Figure 6, we can also observe that simpler OODs like SVHN, DTD, and Pure Color have flatter efficiency gain curves than more complex OODs like LSUN and Tiny ImageNet. This is because simpler OODs are assigned high OOD probabilities and hence are less susceptible to ξ changes.

The voting hyperparameter *k* is also pivotal in controlling the performance-cost trade-off. A higher *k* value means more positive votes an input needs before being identified early as OOD, thereby reducing the number of inputs stopped early. As *k* increases, the framework will eventually become its non-early-stopping counterpart.

**Figure 7:**
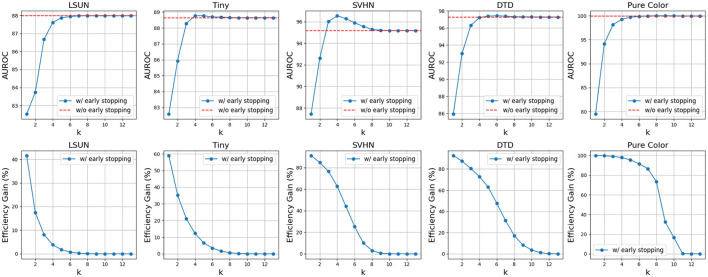
Trends of AUROC and efficiency gain as voting count *k* increase using VGG-16 backbone and CIFAR10 InD.

**Figure 8:**
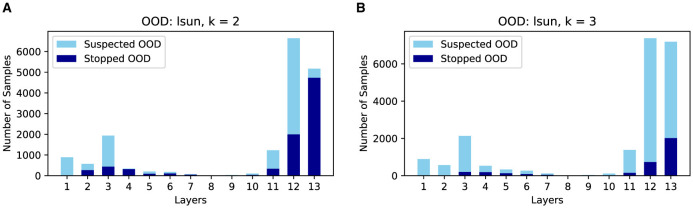
Number of suspected/early stopped LSUN OODs at different layers of VGG-16 using CIFAR10 InD. **(A)** k = 2. **(B)** k = 3.

**Figure 9:**
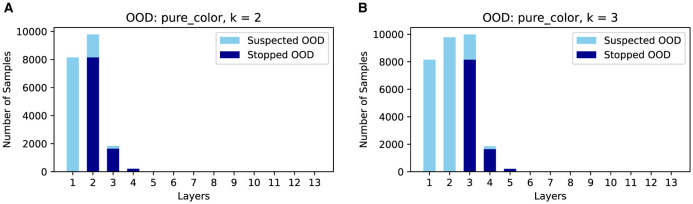
Number of suspected/early stopped Pure Color OODs at different layers of VGG-16 using CIFAR10 InD. **(A)** k = 2. **(B)** k = 3.

As shown in Figure 7, there is a clear trade-off between AUROC and efficiency gain. Higher *k* values enhance AUROC but reduce efficiency gains, while lower *k* values improve efficiency but may degrade AUROC. Moreover, as shown in Figure 8, complex OODs such as LSUN are mostly flagged as suspected OODs (i.e., having OOD probabilities greater than the predefined threshold ξ) at the ending layers. Increasing *k* will postpone the early stopping to the latter layers, allowing more opinions from different OOD detectors to be considered for more accurate detection. A similar effect is observed for simple OODs, such as the Pure Color images, as shown in Figure 9, where with *k* increasing from 2 to 3, most Pure Color OODs are stopped at layer 3 instead of layer 2. From Figure 7 we also observe that efficiency gains are most pronounced for simpler OOD datasets such as SVHN, DTD, and Pure Color, in contrast to more complex datasets like LSUN and Tiny ImageNet, indicating that early stopping yields greater computational savings for less complex OODs as a large amount of them can be identified and stopped very early. Additionally, while *k* is crucial for achieving a satisfactory balance between performance and cost, the trends show that AUROC maintains a relatively flat curve with increasing *k* values after a certain point (*k*≥3 in this case). This demonstrates that although a very small *k* can drastically improve efficiency, it comes at a great cost to OOD detection accuracy, but as we increase the *k* value, ES-OOD's OOD detection performance remains robust against variations in *k* within a large range.

### 4.6 Ablation study

Here, we compare the OOD detection performance of the proposed ES-OOD framework with each of its individual OOD detectors on a mixed OOD dataset containing five OOD datasets (LSUN, Tiny ImageNet, SVHN, DTD, and Pure color). The results are shown in Table 4. Using VGG-16 as an example, for both CIFAR10 and CIFAR100 InD settings, ES-OOD consistently achieves better performance than any single OOD detector.

**Table 4:** A comparison between the ES-OOD and its individual OOD detectors in the intermediate layers.

**InD/model**	**Metric**	*C*_1_	*C*_2_	*C*_3_	*C*_4_	*C*_5_	*C*_6_	*C*_7_	*C*_8_	*C*_9_	*C*_10_	*C*_11_	*C*_12_	*C*_13_	**ES-OOD**
CIFAR10 VGG-16	AUROC ↑	60.20	78.14	89.55	89.00	83.92	81.13	77.99	70.45	65.96	71.58	83.57	89.20	91.62	**93.73**
		AUPR ↑	89.18	94.60	97.55	97.51	96.36	95.75	94.87	92.80	87.07	89.27	94.46	97.08	97.79	**98.48**
		FPR at 95% TPR ↓	95.47	84.39	61.76	64.55	77.97	85.61	89.12	95.19	88.47	82.01	56.04	63.22	37.16	**28.25**
	CIFAR100 VGG-16	AUROC↑	51.87	70.09	83.71	82.61	79.72	77.08	76.17	69.55	72.43	70.38	62.66	37.80	73.46	**85.34**
		AUPR↑	85.56	91.89	95.93	95.85	95.19	94.51	93.84	91.90	91.11	90.27	86.70	76.10	90.42	**95.93**
		FPR at 95% TPR↓	94.75	89.32	76.22	80.75	83.96	86.80	86.00	90.39	81.74	86.29	85.16	96.38	65.37	**52.58**

Performance has been averaged across all OOD datasets. Evaluation metrics with “↑” indicate higher values are better, while those with “↓” indicate lower values are better. The best values are highlighted in bold.

## 5 Conclusion

In conclusion, we introduced ES-OOD, a novel layer-adaptive OOD detection framework with early stopping. By attaching OOD detectors at intermediate layers and employing a layer-adaptive scoring, ES-OOD can effectively detect OODs with varying complexity at their most suitable layers. The motivation behind our approach stems from the pressing need for efficient and reliable OOD detection in real-time systems, where traditional methods prioritize detection effectiveness but often at the cost of significant computational resources. This limitation is particularly evident in applications like autonomous driving and medical diagnosis. To address these challenges, ES-OOD integrates early stopping and layer-adaptive scoring to minimize computational overhead while maintaining superior detection accuracy. ES-OOD utilizes an early stopping strategy to terminate inference when confident OOD predictions are made at intermediate layers and includes a voting mechanism to ensure the true positive rate. The framework is compatible with any existing DNNs and does not require OOD samples during training. By reducing computational costs by up to 99.1% without compromising on accuracy, ES-OOD effectively strikes a balance between efficiency and accuracy, making it highly suitable for resource-constrained environments in real-world applications. Extensive experiments demonstrate that ES-OOD is significantly faster and more effective than state-of-the-art baselines in detecting OODs across various DNN architectures.

## Data Availability

The original contributions presented in the study are included in the article/supplementary material, further inquiries can be directed to the corresponding author.

## Footnotes

^1^The source code and datasets are available at: https://github.com/haoliangwang86/ES-OOD.
